# The Choice Point Model of Acceptance and Commitment Therapy With Inpatient Substance Use and Co-occurring Populations: A Pilot Study

**DOI:** 10.3389/fpsyg.2021.758356

**Published:** 2021-10-28

**Authors:** Brian M. Berman, Kris Kurlancheek

**Affiliations:** ^1^Retreat Behavioral Health, Department of Psychology, Ephrata, PA, United States; ^2^Retreat Behavioral Health, Clinical Department, Ephrata, PA, United States

**Keywords:** acceptance and commitment therapy, substance use disorder, inpatient, transdiagnostic, behavioral health, mindfulness, choice point

## Abstract

**Objectives:** Acceptance and Commitment Therapy (ACT) is an empirically supported treatment which aims to enhance self-acceptance and a commitment to core values. The present study examined the effectiveness of the Choice Point model of ACT in a residential substance use disorder (SUD) setting. Choice Point is a contemporary approach to ACT and targets transdiagnostic processes.

**Methods:** This uncontrolled quasi-experimental design assessed 47 participants taking part in Choice Point for Substances (CHOPS) in order to investigate its influence on psychological inflexibility, values-based action, and self-compassion over time. The study additionally assessed for sleeper effects and associations between transdiagnostic processes and warning signs of relapse.

**Results:** Findings demonstrated a decrease in psychological inflexibility and increases in values-based action and self-compassion over time. Gains were maintained at follow-up, and sleeper effects were observed for psychological inflexibility and mindfulness. Correlational analysis suggested that all transdiagnostic processes were related to warning signs of relapse at follow-up.

**Conclusion:** These results provide preliminary evidence for the feasibility, acceptability, and effectiveness of CHOPS for SUD. Observed sleeper effects in psychological inflexibility and mindfulness indicate that CHOPS may provide longer-term benefits critical to a population where relapse is common. While encouraging, these findings should be interpreted with caution. Future research should utilize comparison groups when investigating CHOPS.

## Introduction

According to the Substance Abuse and Mental Health Service Administration (SAMHSA), substance use disorder (SUD) is a nationwide epidemic with approximately 21.5 million adolescents and adults meeting diagnostic criteria (Center for Behavioral Health Statistics and Quality, 2015). The Diagnostic and Statistical Manual of Mental Disorders (DSM-5) defines SUD as the act of continuing to engage in substance use despite negative effects to cognitive, behavioral, and physiological functioning (American Psychiatric Association, 2013). SUD has been shown to impact social, personal, and occupational wellbeing while also contributing to disease, elevated crime rates, and loss of productivity (American Psychiatric Association, 2013; Center for Behavioral Health Statistics and Quality, 2015).

A multitude of factors contribute to the etiology of SUD including psychological, social learning, social-situational influences, and biological predisposition (Witkiewitz et al., 2014; Smith, 2021). These factors are both internal (i.e., personality and affective experience) and external (i.e., familial and peer interaction) in nature and impact the course of SUD (Witkiewitz et al., 2014). Nearly 8 million Americans are also identified as having one or more co-occurring mental health disorders, further contributing to relapse (Center for Behavioral Health Statistics and Quality, 2015; Ii et al., 2019). This suggests that one-third of individuals with SUD also present with comorbidities, such as depression, anxiety, stress, and posttraumatic stress disorder (PTSD; Hermann et al., 2016; Svanberg et al., 2017). Innovative approaches capable of concurrently targeting multiple diagnoses across varied life domains are needed (Roos et al., 2017).

Traditionally, SUD is treated with evidence-based practices, such as cognitive behavioral therapy (CBT), motivational interviewing (MI), and contingency management (CM; Lee et al., 2015). While established protocols have shown to be efficacious for SUD treatment, 30 to 50% of individuals remain abstinent for only short periods of time (Lee et al., 2015; Ii et al., 2019). Due to limitations treating chronic and comorbid presentations, established protocols may not be best suited for long-term SUD treatment (Clarke et al., 2014; Lee et al., 2015). Transdiagnostic approaches which target processes existing across disorders are warranted (Ii et al., 2019).

## Background

Acceptance and Commitment Therapy (ACT) is a transdiagnostic treatment which has increasingly gained in interest over the past few years. Unlike prevailing mechanistic approaches, ACT has its foundations grounded in functional contextualism (Hayes, 2004; Hayes et al., 2012). Contextual approaches, or *process-based therapies*, examine the way in which differing contexts affect the function of behavior (Harris, 2019; Hofmann and Hayes, 2019).

While a primary aim of traditional CBT is to alter the content of thought, ACT works to modify one’s relationship with private events through acceptance and change processes (Zhang et al., 2018; Hofmann and Hayes, 2019). ACT’s primary goal is to increase *psychological flexibility*, or the act of being present with aversive stimuli, while remaining committed to actions consistent with core values (Dindo et al., 2017). This transdiagnostic process is strengthened using six core processes: mindfulness, acceptance, self-as-context, cognitive defusion, committed action, and values (Hayes et al., 2012; Zhang et al., 2018). Together, all six processes work in concert to increase psychological flexibility, which studies indicate may be more effective than symptom reduction approaches (Stotts and Northrup, 2015; Dindo et al., 2017).

ACT theory purports that psychological inflexibility, or responding narrowly to internal states, helps develop and maintain substance use and mental health disorders (Levin et al., 2014). Pervasive patterns of emotional and cognitive avoidance, a primary contributor to psychological inflexibility, restrict values-consistent choices and paradoxically increase unwanted private events (Levin et al., 2014). This *experiential avoidance* is also transdiagnostic, resulting in avoidance of cravings and post-acute withdrawal symptoms which potentially further drug use, relapse, and a neglecting of values (Levin et al., 2014; Stotts and Northrup, 2015). By directly targeting experiential avoidance and psychological inflexibility, ACT aims to alter maladaptive escape strategies, promote experiential acceptance, and create greater flexibility in decision making (Hayes et al., 2012; Dindo et al., 2017). Because psychological inflexibility underlies a variety of disorders, targeting this transdiagnostic process may be critical for creating lasting behavior change in SUD and co-occurring populations.

### Empirical Support for ACT and SUD

ACT is recognized by the SAHMSA and the American Psychological Association (APA) as an *empirically supported treatment* for SUD, depression, mixed anxiety, obsessive compulsive disorder (OCD), and chronic pain (Stotts and Northrup, 2015; Dindo et al., 2017). Since its inception, there have been over 325 randomized controlled trials using ACT (Gloster et al., 2020).

With regards to SUD, ACT was shown to be effective for the treatment of opioid use disorder, cannabis dependency, alcohol use disorder, and nicotine dependency (Luoma et al., 2012). Studies conducted by Shorey et al. (2017) and Stotts et al. (2015) demonstrated the benefit of targeting transdiagnostic processes, such as experiential avoidance, for SUD. Stotts et al. (2015) showed that participants who failed to respond to a traditional CM intervention exhibited higher levels of experiential avoidance. Because no differences were found in the severity of negative affect, impulsivity, or cravings between responders and non-responders, experiential avoidance was presumed the main mediating factor (Stotts et al., 2015). Shorey et al. (2017) found that higher experiential avoidance was significantly related to drug and alcohol cravings in an SUD residential setting.

A number of meta-analyses have also compared ACT to traditional CBT for SUD. Ruiz (2012) found that ACT outperformed all cognitively focused CBT interventions and was potentially more effective at treating co-occurring depression, anxiety, eating disorders, and emotional disorders. A second meta-analysis comparing ACT to alternative treatments for substance use found that ACT was at least as effective as traditional CBT, nicotine replacement therapy, and 12-step approaches, however, was better able to maintain and improve upon abstinence compared to each intervention (Lee et al., 2015). A recent review of ACT meta-analyses was performed and indicated that effect sizes favored ACT for SUD over all other control groups (Gloster et al., 2020).

There is a paucity of research examining transdiagnostic processes and ACT for co-occurring disorders. Meyer et al. (2018) found that an ACT-based intervention significantly reduced comorbid PTSD and alcohol symptoms which were maintained at follow-up. Additional decreases in functional disability, depressive symptoms, and suicidal ideation were also maintained, while symptom changes were associated with reductions in psychological inflexibility and experiential avoidance (Meyer et al., 2018). Similarly, Levin et al. (2014) found that psychological inflexibility was more strongly related to SUD with depression and anxiety than SUD alone, highlighting the significance of targeting psychological inflexibility in co-occurring disorders (Levin et al., 2014).

Additional pilot studies include an investigation by Thekiso et al. (2015) who compared ACT with treatment as usual (TAU) for alcohol use disorder and comorbid affective disorders in a hospital setting. The ACT condition demonstrated significant improvements compared to TAU including increased abstinence from alcohol, fewer depression and anxiety symptoms, and reduced cravings at follow-up. Another study by Heffner et al. (2015) examined the effectiveness of an ACT smoking cessation group for nicotine dependency and co-occurring bipolar disorder. Researchers found that a 50% increase in acceptance was associated with a 51% increase in abstinence and that at least half of participants demonstrated a 50–60% reduction in frequency of smoking.

Where therapy outcomes typically deteriorate with time, the opposite has been observed in several ACT studies. This unique ability to maintain outcomes at follow-up while continuing to exhibit therapeutic benefits has been labeled the *sleeper effect* (Lee et al., 2015). Luoma et al. (2012) demonstrated a similar sleeper effect when comparing ACT with traditional CBT for the treatment of shame in a residential SUD setting. Continuous treatment gains were observed in the areas of shame, substance use, and treatment adherence across the study and at follow-up (Luoma et al., 2012). In a population where relapse is common, interventions capable of building upon therapeutic gains are needed. Additional investigations into the relationship between transdiagnostic processes and warning signs of relapse may also prove beneficial as warning signs are a significant predictor of future substance use (Miller and Harris, 2000).

### ACT and Self-Compassion

It is intuitive that self-compassion be applied to SUD as substances are often used to avoid shame and self-criticism, while self-compassion targets the biological threat system which gives rise to both (Gilbert, 2014; Luoma et al., 2019). Self-compassion was described by Neff and Tirch (2013) as a combination of self-kindness, common humanity, and mindfulness. Research investigating self-compassion for SUD is in its early stages. One investigation demonstrated the way in which patients at a residential SUD facility increased self-compassion while reducing guilt and shame after a 4-week self-compassion intervention (Held et al., 2018). Phelps et al. (2018) found that lower self-compassion was associated with a higher risk of SUD, while Platt et al. (2018) indicated that self-compassion interventions produced speedier reductions of daily cigarette smoking.

As a treatment approach aiming to foster self-acceptance, perspective taking, and mindfulness, ACT may be particularly well suited for developing self-compassion (Yadavaia et al., 2014). First, by building an awareness of the *observer self*, a self which mindfully observes the occurrence of private events, contextual changes make it possible to relate to the self in a kinder, more compassionate way (Hayes et al., 2012; Yadavaia et al., 2014). According to Relational Frame Theory (RFT), a science of language and cognition underlying ACT, *deictic relational framing* allows for this perceptual shift to occur (Neff and Tirch, 2013; Yadavaia et al., 2014). *Deictic framing* can be defined as a way in which human language helps foster a sense of self and perspective taking through derived relationships with others (Hayes et al., 2012). Through deictic framing, relationships are derived between *I/you*, *here/there*, and *now/then*. When applied interpersonally, deictic framing creates the context in which common humanity functions. When applied internally, deictic framing allows for intrapersonal shifts in context, which are necessary for responding to private events with compassion.

Second, ACT promotes acceptance of internal states while committing to values-consistent decision making. This psychologically flexible state is inherently self-compassionate as it encourages mindfulness, self-kindness, and movement toward universal values (Neff and Tirch, 2013; Yadavaia et al., 2014; Ong et al., 2019). While limited in scope, research has shown ACT to be an efficacious intervention for enhancing self-compassion. Yadavaia et al. (2014) found that after only three workshops, ACT significantly increased self-compassion at post-treatment and follow-up. Effect sizes were comparable to traditional self-compassion protocols, but the ACT intervention was shorter in duration (Yadavaia et al., 2014).

### The Choice Point Model of ACT

The primary aim of Choice Point is to minimize narrow or inflexible behavior by increasing values-consistent choices (Harris, 2017). This is accomplished through building an awareness of *choice points*, or moments in time when a person is faced with making life choices that are values-consistent or values-inconsistent. Through increased awareness of choice points, individuals are better able to lessen reactivity to internal states, allowing for enhanced flexibility and committed action (Harris, 2017). Identifying choice points may also have the added benefit of strengthening resilience (Gervis and Goldman, 2020).

Where traditional ACT utilizes six core processes to increase psychological flexibility and reduce experiential avoidance, Choice Point aims to simplify this approach and create a user-friendly experience (Ciarrochi et al., 2013). Using middle-level terms, such as *toward moves*, *away moves*, *hooks, values*, and *choice points*, the Choice Point model provides a conceptual overview which is easy for patient consumption (Harris, 2019). Choice Point differs from standard ACT in three distinct ways. First, the Choice Point model targets movements toward and away from values, in contrast to toward values and away from pain (Harris, 2017). This allows for the model to target a broader range of inflexible behaviors regardless of appetitive or aversive control (Harris, 2017). Second, building awareness of choice points in order to increase values-consistent behaviors is unique to the Choice Point approach (Ciarrochi et al., 2013). Third, the Choice Point model overtly identifies self-compassion as a value, which is not typical of traditional ACT (Ciarrochi et al., 2013).

### Choice Point Applied to Substance Use Disorder

SUD has a multitude of factors contributing to its complexity including psychological, social learning, and biological predispositions (Witkiewitz et al., 2014). Within the context of these proximal and distal factors, traditional ACT and the Choice Point model both help individuals move toward values at times when committed action is difficult. However, Choice Point ACT may be particularly well suited for SUD because of the way in which it targets both external and internal factors contributing to the development and reinforcement of the disorder. Individuals often come under appetitive control when reproducing rewarding stimuli, while falling under aversive control when avoiding unpleasant stimuli (Wilson, 2009). By increasing opportunities for values-consistent choices, choice point awareness may disrupt external reinforcement, such as social learning or maladaptive pleasure-seeking behaviors, and instead enhance values-driven appetitive control. Additionally, choice point awareness may disrupt experiential avoidance of drug cravings, emotional pain, and other internal private events specific to the individual. Utilizing choice points to alter both external reinforcement and internal avoidance patterns allows for broader, more flexible behavioral repertoires for those with SUD (Harris, 2017). This is perhaps the most significant benefit of applying Choice Point as an alternative to standard ACT for SUD.

### Aim of the Present Study

This pilot study aims to determine the effectiveness of Choice Point for Substances (CHOPS) at influencing transdiagnostic processes in an inpatient SUD setting. CHOPS is a manualized approach to the Choice Point model of ACT specifically for SUD (Berman, 2017, Unpublished Manual). Like the Choice Point model, CHOPS simplifies ACT middle-level terms, builds awareness of choice points, and directly targets self-compassion as a value. Therapeutic activities, group-format, session length, and session frequency were all tailored for use in an inpatient setting.

It was hypothesized that 16 sessions of CHOPS would impact transdiagnostic processes in three ways: (a) psychological inflexibility would reduce over time, while values-based action and self-compassion would increase over time, (b) sleeper effects would be observed for psychological inflexibility, values-based action, and self-compassion, and (c) psychological inflexibility would be positively associated with warning signs of relapse, while values-based action and self-compassion would be negatively related to relapse signs at follow-up. To our knowledge, this is the first investigation into the effectiveness of a manualized approach to the Choice Point model in a residential SUD setting.

## Materials and Methods

### Participants

Recruitment occurred at a 30-day residential SUD treatment facility located in Pennsylvania. A total of 115 participants initially signed consent to take part in the study. Due to high attrition rates resulting mainly from insurance denials, employment obligations, and family needs, 59 participants (51%) left the facility prior to treatment completion. High attrition is common in SUD settings, and rates were comparable with those found in previous studies (Roseborough et al., 2015; Dindo et al., 2017; Svanberg et al., 2017). Seven additional participants (6%) withdrew from the study citing a desire to participate in TAU instead of the study group. Two participants (1.7%) left the facility against medical advice (AMA). One of these participants left prior to intervention involvement, while the other left shortly after participation began. These data suggest that while attrition was generally high, only 6% of participant decidedly withdrew from the study. Additionally only 1% of active participants left treatment AMA which was discovered to be well below facility AMA rates.

All participants were between 18 and 66years of age with a mean age range of 18–34years old (see Table 1). There were a larger number of male participants (63.8%) than female participants (36.2%) who completed the intervention (*N*=47). Each participant met criteria for one or more SUDs. Of the 47 participants who completed the study, almost half (48.9%) self-reported alcohol as their primary used substance. Opioid use (25.5%), polysubstance use (17.0%), stimulant use (4.3%), and anxiolytic/hallucinogenic use (4.2%) were also reported as primary reasons for admission. The majority of participants (93.6%) additionally self-identified as having one or more co-occurring mental health disorder. Anxiety (12.8%), depression (6.4%), and chronic pain (2.1%) were reported as main co-occurring disorders among participants. The highest prevalence of co-occurring disorders presented as anxiety with depression (57.4%) or a combination of anxiety, depression, and chronic pain (14.9%).

**Table 1:** Sociodemographic characteristics of participants.

Characteristic	*n*	%	Characteristic	*n*	%
Gender			Employment		
Male	30	63.8	Full-Time	26	55.3
Female	17	36.2	Part-Time	6	12.8
Transgender	0	0	Unemployed	15	31.9
Other	0	0	Income		
Age			$0–$30,000	9	19.1
18–24	9	19.1	$31,000–$70,000	18	38.3
25–34	12	25.5	$71,000–$100,000	8	17.0
35–44	8	17.0	$100,000+	12	25.5
45–54	10	21.3	Education		
55–64	7	14.9	High School	21	44.7
65+	1	2.1	GED	5	10.6
Marital status			College Diploma	12	25.5
Single	19	40.4	Master’s Degree	3	6.4
Married	12	25.5	Doctoral Degree	3	6.4
Divorced	9	19.1	Other	3	6.4
Separated	6	12.8	Drug of Choice		
Other	1	2.1	Opiates/Heroin	12	25.5
Ethnicity			Alcohol	23	48.9
African American	3	6.4	Stimulants	2	4.3
Caucasian	41	87.2	Hallucinogens	1	2.1
Hispanic	1	2.1	Anxiolytics	1	2.1
Asian	1	2.1	Other^a^	8	17.0
Other	1	2.1	Mental Health Difficulty		
Religion			None	3	6.4
Christian	31	66.0	Depression	3	6.4
Buddhist	1	2.1	Anxiety	6	12.8
Islam	0	0	Depression and Anxiety	27	57.4
Judaism	0	0	Chronic Pain	1	2.1
Other	15	31.9	Chronic Pain with Depression and Anxiety	7	14.9

*n=47*.

a *When participants indicated more than one drug of choice it was coded as “other.”*

### Procedure

This study was approved by the Lancaster General Health Institutional Review Board (IRB). Participants took part in a 16-session manualized group intervention over the course of 4weeks. Sessions took place four times per week and were each 1h in length. Closed groups were conducted over a 9-month period with 3-month subsequent follow-up assessments. One primary facilitator (Bachelor’s level clinician) implemented the manual while a secondary group leader acted as an ancillary therapist. On one occasion, a third clinician facilitated the group when both primary and secondary clinicians were unavailable. In order to enhance study fidelity, each clinician received direct manual training with a doctoral level psychologist specializing in ACT. Study fidelity was further strengthened using an intervention checklist in order to rate facilitators during each group session and assure accurate manual implementation. The intervention checklist is an unstandardized checklist developed for this study in order to assess facilitators in seven main areas of focus: (1) group start time, (2) review of middle-level terms, (3) psychoeducation, (4) appropriate implementation of therapeutic activity, (5) processing of activity, (6) clinician engagement and enthusiasm, and (7) preparation and knowledge regarding the material. Checklist ratings indicated that facilitators adhered to the protocol, were competent in their implementation, and were free of therapy contamination.

All eligible patients were provided the opportunity for study participation during admission. Inclusion criteria required participants be English speaking, admitted as an inpatient resident, and 18years of age or older. Patients were excluded if they presented with significant cognitive impairment, or previously attended Choice Point groups. New patients were recruited in 4-week intervals. Those meeting inclusion criteria were invited to attend an informed consent meeting where they were educated about the purpose of the study, as well as the risks and benefits to their participation.

At the informed consent meeting, all attendees were greeted with incentives limited to food and refreshments. No additional incentives were provided. Informed consent and private health information (PHI) forms were reviewed orally and participants provided written consent. A demographics form and three assessment measures were also completed by participants. Those unable to attend due to scheduling conflicts met with researchers individually. Precautions to confidentiality were taken including de-identifying subject names and securing documentation behind multiple locked doors.

Thirty-one participants provided 3-month follow-up data which were obtained through phone, email, Internet, and in-person collection. When corresponding through email, participants received a link for completing assessments through Survey Monkey. Those who were reached by phone were provided the option to complete assessments through telecommunication or to have hard copies mailed to their home. If the participant was unable to be reached by email or phone, a voice message was left when permitted. Participants who preferred to complete assessments in person were arranged to have face-to-face meetings.

### Treatment Protocol

#### Manual Overview

CHOPS is a manualized approach to the Choice Point model specifically designed for a residential SUD setting (Berman, 2017, Unpublished Manual). All 16 sessions were similar in format: (a) review of choice point middle-level terms, (b) psychoeducation, (c) therapeutic/experiential exercise, and (d) therapeutic processing. Psychoeducational worksheets were disseminated on corresponding days. CHOPS was heavily informed by Ciarrochi et al. (2013) unpublished PowerPoint introduction to the Choice Point model of ACT. CHOPS was developed due to the absence of an existing Choice Point protocol for substance use.

CHOPS combined traditional Choice Point interventions, such as the *choice point diagram*, with Choice Point consistent exercises, such as *Wise Choices* activities (Morton and Shaw, 2012). Investigators also utilized traditional ACT exercises as well as novel Choice Point interventions. Examples of traditional ACT exercises included *values assessment*, *milk* exercise, *tin can monster*, and *leaves on a stream*. Novel Choice Point interventions included *hook sorting* tasks and the *toward/away chair*.

CHOPS incorporated self-compassion exercises with the aim of increasing *covert self-compassion* and *overt self-compassion*. Covert self-compassion was defined as the process of self-acceptance, self-validation, and self-kindness occurring intrinsically during times of psychological flexibility. Overt self-compassion was defined as a purposeful act of valuing self-compassion while taking steps in pursuit of that value. Covert self-compassion was cultivated through the use of mindfulness interventions, acceptance exercises, and a commitment to core values. Overt self-compassion was targeted using the Choice Point diagram to identify compassion-focused values.

Additionally, self-compassion exercises were utilized for the purpose of fostering shifts in perspective taking. Participants imagined speaking compassionately to a friend, followed by a shift in perspective toward the self. Altering between *I/you* and *here/there* perspectives aimed to strengthen deictic framing processes necessary for self-compassion.

#### Manual Implementation

Session 1 was an introduction to the Choice Point model. Participants were exposed to middle-level terms, a Choice Point diagram, and an introductory video. Sessions 2 and 3 helped participants to clarify values while labeling internal and external obstacles common to SUD (referred to as hooks). Sessions 4 through 6 provided psychoeducation about mindfulness skills and how to apply those skills to cravings, affective hooks, and making wise choices. Specifically, participants took part in mindfulness of breath, mindful hook sorting tasks, and a tin can monster meditation.

Sessions 7 and 8 educated participants about toward/away moves using the *bus metaphor* and patterns of experiential avoidance using an *avoidance loop* activity. These demonstrated the manner in which habitual avoidance of cravings, unwanted affect, and cognition negatively impacts values-based decision making. Session 9 saw participants identifying choice points by categorizing values, toward moves, away moves, and hooks into Choice Point diagrams. Sessions 10 and 11 introduced cognitive defusion skills where participants practiced mindfully unhooking from the content of thought while concurrently observing the process of thought (observer self). Session 12 aimed to help participants practically apply choice points through taking *BOLD* action. Participants practiced *breathing* slowly, *observing* internal and external experience, *listening* to their values, and *deciding* to make values-consistent choices. Sessions 13 through 16 helped participants identify variations in self-compassion while sorting them into Choice Point diagrams. Participants additionally engaged in experiential exercises meant to strengthen deictic framing and self-compassion through perspective taking.

### Measures

Participants completed four separate assessment measures: Acceptance and Action Questionnaire–II (AAQ-II), Valued Living Questionnaire (VLQ), Self-Compassion Scale (SCS), and the Advanced Warning of Relapse Questionnaire–Revised (AWARE). Because a large number of participants were expected to present with co-occurring mental health disorders, the Acceptance and Action Questionnaire–Substance Abuse (AAQ-SA) was not chosen due to its craving-specific items. The AAQ-II, VLQ, and SCS were administered before Session 1, after Session 8, after Session 16, and again at 3-month follow-up. AWARE was also administered at 3-month follow-up. Feasibility and acceptability were measured by assessing treatment adherence, therapeutic outcomes, recruitment success, and self-reported patient satisfaction.

#### Acceptance and Action Questionnaire–II

The AAQ-II is a 7-item self-report measure of psychological inflexibility and experiential avoidance (Miron et al., 2015). Higher scores on the 7-point Likert scale are indicative of greater psychological inflexibility and experiential avoidance (Dixon et al., 2016). The AAQ-II has good internal consistency (*α*=0.84), appropriate discriminative validity, and strong test-retest reliability (Bond et al., 2011; Miron et al., 2015).

#### Valued Living Questionnaire

The VLQ is a two-part questionnaire with 10 items in each section. Part one assesses values *importance* and asks individuals to rate the importance of values in 10 specific valued-life domains (Wilson et al., 2010). Part two assesses the *consistency* with which values-consistent actions occurred during the past week. Both assessment sections are scored on a 10-point Likert scale and are used to calculate a valued living composite score. Higher composite scores are representative of increased values-based action. The Importance and Consistency subscales showed good (*α*=0.83) and adequate (*α*=0.60) internal consistency. The Valued Living composite also demonstrated adequate internal consistency (*α*=0.74). The VLQ is correlated with measures of depression and experiential avoidance and displays adequate internal and temporal consistency (Wilson et al., 2010; Dixon et al., 2016).

#### Self-Compassion Scale

The SCS is a 26-item scale which measures trait levels of self-compassion (Neff, 2015). Scored on a 5-point Likert scale, the SCS has demonstrated good overall psychometrics and creates a self-compassion total score by calculating the mean of six subscale mean scores. Subscales include self-kindness, self-judgment, common humanity, isolation, mindfulness, and over-identification (Neff, 2015; Neff et al., 2017). The SCS demonstrated excellent internal consistency (*α*=0.92), good convergent and discriminate validity, and strong predictive validity (Neff, 2003, 2015).

#### Advanced Warning of Relapse Questionnaire–Revised

AWARE has 28 items and is scored on a 7-point Likert scale. The self-report measure assesses warning signs of alcohol relapse, with higher scores indicative of greater relapse signs (Miller et al., 1996). AWARE has been shown to be a good predictor of relapse occurrences. It has demonstrated excellent internal consistency (*α*=0.92) and good test-retest reliability (Miller and Harris, 2000). For purposes of this study, phrasing was refined to represent signs of relapse more generally rather than alcohol-specific relapse.

### Design

Using a quasi-experimental design, the current study aimed to examine the effectiveness of CHOPS in an inpatient SUD setting. *A priori* power analysis was performed using G^*^Power in order to calculate sample size and power for repeated measures ANOVA, dependent *t*-test, and Pearson r correlational analyses (Faul et al., 2007). Analyses indicated that sample sizes consisting of 28, 34, and 64 participants, respectively, were required for 80% power. The main analysis exhibited adequate power while follow-up analyses including *t*-tests and correlational analysis were underpowered.

Forty-seven participants located in an inpatient SUD facility completed the 16-session group intervention. A one-way repeated measures ANOVA was performed on multiple occurrences in order to assess change in psychological inflexibility, values-based action, and self-compassion over three points in time (pre-treatment, mid-treatment, and post-treatment). Pre-treatment data were collected prior to Session 1, mid-treatment data after Session 8, and post-treatment data after Session 16.

Paired sample *t*-tests were also performed comparing baseline functioning with 3-month follow-up (*n*=30) and post-treatment functioning with 3-month follow-up (*n*=20) for transdiagnostic processes. Due to the frequency of early discharge, participants who completed a minimum of 8 sessions were included in follow-up data resulting in varied samples sizes. Twenty-nine participants (*n*=29) were additionally assessed using a bivariate correlational analysis to determine the extent to which warning signs of relapse were related to psychological inflexibility, values-based action, and self-compassion at 3-month follow-up. Because group attendance was the primary measure of treatment adherence, attendance records were utilized to assess treatment adherence and its relationship with psychological inflexibility, values-based action, and self-compassion post-treatment. Missing data were not present during pre-treatment, mid-treatment, or post-treatment assessments; however, incomplete follow-up data were analyzed using Listwise deletion. Utilizing this analysis contributed to additional variations in sample sizes for *t*-tests and correlational analyses.

## Results

### Main Analysis: Within-Group Comparisons

Three independent repeated measures ANOVA was performed on psychological inflexibility, values-based action, and self-compassion (see Table 2). The Shapiro-Wilk test in combination with p-plot observations was used to test for normality and indicated that all three variables were normally distributed, *p*>0.05. Three significant main effects were observed with a large effect size for each variable. A decrease in psychological inflexibility, *F*(2, 92)=29.89, *p*<0.001, *η^2^*=0.39, was seen across the intervention over time. The Huynh-Feldt correction was used for the VLQ due to violating sphericity, *χ^2^*(2)=10.81, *p*=0.004, and indicated a significant increase in values-based action, *F*(1.70, 78.27)=74.05, *p*<0.001, *η^2^*=0.62. A main effect in self-compassion, *F*(2, 92)=28.21, *p*<0.001, *η^2^ =* 0.38, was also observed over time. All VLQ and SCS subscales were significant, *p*<0.001 (see Table 2).

**Table 2:** Repeated measures ANOVA for psychological inflexibility, values-based action, and self-compassion.

Measure	Pre-treatment		Mid-treatment		Post-treatment		ANOVA	
	M	SD	M	SD	M	SD	*F* ratio	*df*
AAQ-II								
Psychological Inflexibility	35.21	8.47	28.64	10.03	23.85	8.69	29.89^***^	2,92
VLQ								
Valued Living Composite^a^	34.89	17.41	53.38	20.04	64.14	19.70	74.05^***^	1.70,78.27
Values Importance	6.79	1.62	7.36	1.62	7.86	1.48	18.66^***^	2,92
Values Consistency^a^	4.94	1.86	6.82	1.71	7.87	1.51	63.89^***^	1.80,82.82
SCS								
Self-Compassion Total	2.34	0.56	2.80	0.76	3.23	0.81	28.21^***^	2,92
Self-Kindness^a^	2.15	0.69	2.54	0.83	3.06	1.00	17.67^***^	1.82,83.81
Self-Judgment^a^	3.69	0.85	3.25	0.84	2.91	0.93	14.23^***^	1.71,78.65
Common Humanity	2.46	0.86	2.96	0.99	3.29	0.95	17.66^***^	2,92
Isolation	3.74	0.81	3.14	0.96	2.72	0.93	32.07^***^	2,92
Mindfulness	2.52	0.73	2.88	0.92	3.34	0.86	18.14^***^	2,92
Over-identification	3.64	0.80	3.21	0.86	2.69	0.89	23.64^***^	2,92

*n=47. ANOVA, Analysis of Variance. AAQ-II, Acceptance and Action Questionnaire–II; VLQ*, *Valued Living Questionnaire; and SCS*, *Self-Compassion Scale*.

a *The Huynh-Feldt correction was used for valued living, values consistency, self-kindness, and self-judgment*.

*** *p<0.001.*

The Bonferroni method was employed to compare means across levels of the intervention (see Table 2). Findings demonstrated decreases in psychological inflexibility and increases in values-based action and self-compassion when comparing pre-treatment with mid-treatment means (*p*<0.001) and pre-treatment with post-treatment means (*p*<0.001). Mid-treatment and post-treatment comparisons were also significant for psychological inflexibility (*p*=0.007), values-based action (*p*<0.001), and self-compassion (*p*=0.001).

### Treatment Maintenance

Paired sample *t*-tests were performed comparing baseline and follow-up functioning in order to assess for treatment maintenance (see Table 3). Results indicated that therapeutic gains were maintained for psychological inflexibility, *t*(29)=10.25, *p*<0.001, *d*=1.87, values-based action, *t*(29)=−5.12, *p*<0.001, *d*=0.94, and self-compassion, *t*(29)=−6.40, *p*<0.001, *d*=1.17, when comparing baseline and follow-up. VLQ and SCS subscales additionally demonstrated significant mean differences (*p*<0.05; see Tables 3, 5).

**Table 3:** Paired sample *t*-test: Treatment maintenance for psychological inflexibility, values-based action, and self-compassion.

Measure	Pre-treatment			Follow-up			*t* score	*df*	*p*
	Mean	SD	S.E. Mean	Mean	SD	S.E. Mean			
AAQ-II									
Psychological Inflexibility	35.97	7.61	1.39	19.03	7.11	1.30	10.25	29	0.000
VLQ									
Valued Living Composite	38.55	16.50	3.01	59.57	19.11	3.49	−5.12	29	0.000
Values Importance	7.20	1.45	0.26	7.81	1.50	0.27	−2.15	29	0.040
Values Consistency	4.96	1.78	0.32	7.09	1.73	0.32	−4.78	29	0.000
SCS									
Self-Compassion Total	2.26	0.69	0.13	3.50	0.96	0.18	−6.40	29	0.000
Self-Kindness	2.13	0.86	0.16	3.32	1.07	0.19	−5.47	29	0.000
Self-Judgment	3.80	0.88	0.16	2.67	0.97	0.18	5.15	29	0.000
Common Humanity	2.33	0.88	0.16	3.56	1.00	0.18	−6.17	29	0.000
Isolation	3.75	0.91	0.17	2.48	1.20	0.22	5.06	29	0.000
Mindfulness	2.43	0.86	0.16	3.69	1.08	0.20	−5.66	29	0.000

*n=30. AAQ-II*, *Acceptance and Action Questionnaire–II; VLQ, Valued Living Questionnaire; and SCS*, Self-Compassion Scale. Over-identification, a subscale of SCS, was assessed using the Wilcoxon Signed Rank Test (see Table 5).

### Sleeper Effect

Paired sample *t*-tests were also performed comparing post-treatment and follow-up to determine if any sleeper effects occurred (see Table 4). Findings indicated significant improvements in psychological inflexibility, *t*(19)=3.29, *p*=0.004, *d*=0.74, from post-treatment to follow-up. Overall self-compassion was not found to be significant, *t*(19)=−1.48, *p*=0.155, when comparing post-treatment and follow-up; however, findings indicated significant improvements in mindfulness, *t*(19)=−2.25, *p*=0.036, *d*=−0.50, an SCS subscale. The Wilcoxon Signed Rank Test was used to compare post-treatment and follow-up for the VLQ. No significant increases in values-based action were observed, *z*=−0.97, *p*=0.332 (see Table 5).

**Table 4:** Paired sample t-test: Sleeper effect for psychological inflexibility and self-compassion.

Measure	Post-treatment			Follow-up			*t* score	*df*	*p*
	Mean	SD	S.E. Mean	Mean	SD	S.E. Mean			
AAQ-II									
Psychological Inflexibility	25.00	6.90	1.54	18.80	7.51	1.68	3.29	19	0.004
SCS									
Self-Compassion Total	3.31	0.71	0.16	3.59	0.83	0.18	−1.48	19	0.155
Self-Kindness	3.17	0.92	0.21	3.51	0.89	0.20	−1.54	19	0.141
Self-Judgment	2.91	0.80	0.18	2.71	0.99	0.22	0.85	19	0.407
Common Humanity	3.45	0.85	0.19	3.79	0.84	0.19	−1.45	19	0.163
Isolation	2.63	0.78	0.18	2.41	1.05	0.23	0.89	19	0.386
Mindfulness	3.41	0.73	0.16	3.84	0.93	0.21	−2.25	19	0.036
Over-identification	2.66	0.75	0.17	2.50	1.05	0.23	0.76	19	0.457

*n=20. AAQ-II*, *Acceptance and Action Questionnaire–II; SCS, Self-Compassion Scale. The Wilcoxon Signed Rank Test was utilized to assess the VLQ total score and VLQ subscale scores (see* **Table 5***).*

**Table 5:** Wilcoxon Signed Rank Test: Treatment maintenance and sleeper effect for self-compassion and values-based action.

Measure	*n*	Pre	Post	*z* score	*p*
		Median	Median		
SCS					
Over-identification^a^	30	4.00	2.25	−3.90	0.000
VLQ					
Valued Living^a^	20	68.80	73.35	−0.97	0.332
Values Importance^b^	20	8.25	8.45	−0.02	0.985
Values Consistency^b^	20	8.35	8.10	−1.49	0.136

*VLQ, Valued Living Questionnaire; SCS, Self-Compassion Scale. Wilcoxon Signed Rank Test was utilized for total scores and subscales violating Shapiro-Wilk test of normality when comparing pre- and post-measurements*.

a Pre- and post-comparisons assessed for “Treatment Maintenance.” Data labeled as “Pre” refer to baseline data and “Post” refer to 3-month follow-up data.

b Pre- and post-comparisons assessed for “Sleeper Effects.” Data labeled as “Pre” refer to post-treatment data and “Post” refer to 3-month follow-up data.

### Relapse Prevention

A bivariate correlational analysis was performed to determine the extent which psychological inflexibility, values-based action, and self-compassion were related to warning signs of relapse at follow-up. Results showed a significant association between warning signs of relapse and transdiagnostic processes, *p*<0.01. Self-compassion and psychological inflexibility exhibited the strongest associations with self-compassion demonstrating an inverse relationship with warning signs of relapse, *r*(27)=−0.68, *p*<0.001, and psychological inflexibility showing a positive relationship with warning signs of relapse, *r*(27)=0.66, *p*<0.001. Values-based action was also negatively associated with warning signs of relapse *r*(27)=−0.58, *p*=0.001. SCS and VLQ subscales, with the exception of the importance subscale, were significant, *p*<0.05 (see Table 6).

**Table 6:** Descriptive statistics and correlations for warning signs of relapse, psychological inflexibility, values-based action, and self-compassion.

Measure	*M*	*SD*	1	2	3	4	5	6	7	8	9	10	11	12
1. Warning Signs of Relapse	72.00	24.16	—											
2. Psychological Inflexibility	19.38	6.98	0.66^***^	—										
3. Valued Living	58.93	19.12	−0.58^**^	−0.41^*^	—									
4. Self-Compassion	3.45	0.95	−0.68^***^	−0.60^**^	0.51^**^	—								
5. Values Importance^a^	7.78	1.52	−0.31	−0.03	0.74^***^	0.47^*^	—							
6. Values Consistency^a^	7.06	1.75	−0.60^**^	−0.44^*^	0.93^***^	0.44^*^	0.52^**^	—						
7. Self-Kindness^b^	3.27	1.05	−0.54^**^	−0.45^*^	0.54^**^	0.95^***^	0.57^**^	0.45^*^	—					
8. Self-Judgement^b^	2.72	0.93	0.60^**^	0.63^***^	−0.32	−0.85^***^	−0.21	−0.29	−0.74^***^	—				
9. Common Humanity^b^	3.54	1.01	−0.44^*^	−0.24	0.44^*^	0.79^***^	0.50^**^	0.34	0.85^***^	−0.53^**^	—			
10. Isolation^b^	2.53	1.19	0.70^***^	0.62^***^	−0.45^*^	−0.93^***^	−0.38^*^	−0.40^*^	−0.85^***^	0.79^***^	−0.60^**^	—		
11. Mindfulness ^b^	3.65	1.07	−0.57^**^	0.49^**^	0.56^**^	0.91^***^	0.56^**^	0.43^*^	0.89^***^	−0.73^***^	0.74^***^	−0.78^***^	—	
12. Over-identification^b^	2.53	1.15	0.73^***^	0.70^***^	−0.38^*^	−0.87^***^	−0.30	−0.39^*^	−0.74^***^	0.73^***^	−0.52^**^	0.89^***^	−0.71^***^	—

*n*=29.

a Valued Living Questionnaire (VLQ) subscale.

b Self-Compassion Scale (SCS) subscale.

* *p*<0.05;

** *p*<0.01;

*** *p*<0.001.

**Table 7:** Descriptive statistics and correlations for treatment adherence, psychological inflexibility, values-based action, and self-compassion.

Measure	*M*	*SD*	1	2	3	4	5	6	7	8	9	10	11	12
1. Treatment Adherence	5.51	0.98	—											
2. Psychological Inflexibility	23.85	8.69	−0.34^*^	—										
3. Valued Living	64.14	19.70	−0.01	−0.35^*^	—									
4. Self-Compassion	3.23	0.81	0.10	−0.64^***^	0.38^**^	—								
5. Values Importance^a^	7.86	1.48	0.01	−0.43^**^	0.86^***^	0.51^***^	—							
6. Values Consistency^a^	7.87	1.51	−0.10	−0.13	0.87^***^	0.13	0.53^***^	—						
7. Self-Kindness^b^	3.06	1.00	0.13	−0.50^***^	0.36^*^	0.90^***^	0.48^**^	0.13	—					
8. Self-Judgement^b^	2.91	0.93	−0.11	0.72^***^	−0.37^*^	−0.90^***^	−0.50^***^	−0.12	−0.70^***^	—				
9. Common Humanity^b^	3.29	0.95	0.01	−0.43^**^	0.24	0.87^***^	0.35^*^	0.06	0.84^***^	−0.66^***^	—			
10. Isolation^b^	2.72	0.93	0.01	0.64^***^	−0.32^*^	−0.84^***^	−0.45^**^	−0.10	−0.63^***^	0.88^***^	−0.59^***^	—		
11. Mindfulness^b^	3.34	0.86	0.22	−0.42^**^	0.28	0.81^***^	0.34^*^	0.11	0.83^***^	−0.56^***^	0.79^***^	−0.46^**^	—	
12. Over-identification^b^	2.69	0.89	−0.07	0.65^***^	−0.39^**^	−0.90^***^	−0.53^***^	−0.13	−0.70^***^	0.88^***^	−0.68^***^	0.82^***^	−0.60^***^	—

*n*=47.

a Valued Living Questionnaire (VLQ) subscale.

b Self-Compassion Scale (SCS) subscale.

* *p*<0.05;

** *p*<0.01;

*** *p*<0.001.

### Treatment Adherence

Attendance was analyzed as a measure of treatment adherence, intervention feasibility, and intervention acceptability. A bivariate correlational analysis was performed in order to determine the extent to which psychological inflexibility, values-based action, and self-compassion were related to treatment adherence post-treatment. Findings indicated that psychological inflexibility demonstrated a moderate inverse association with treatment adherence, *r*(45)=−0.34, *p*=0.019. Values-based action and self-compassion were not significantly correlated with treatment adherence post-treatment, *r*(45)=0.01, *p*=0.965, *r*(45)=0.10, *p*=0.502, respectively.

## Discussion

The present study aimed to assess the effectiveness of CHOPS protocol at improving psychological inflexibility, values-based action, and self-compassion in a residential SUD setting. Also examined was the extent to which warning signs of relapse were associated with all three transdiagnostic processes at 3-month follow-up. Further, data were analyzed to determine the relationship between treatment adherence and psychological inflexibility, values-based action, and self-compassion post-treatment.

Established treatment approaches for SUD have demonstrated limited short-term and long-term success (Ii et al., 2019). However, present findings showed significant overall improvements in psychological inflexibility, values-based action, and self-compassion indicating that participants were more willing to experience unwanted internal events, engage in actions consistent with values, and treat themselves more compassionately post-treatment. In other words, participants were more accepting of thoughts and feelings, made choices consistent with values, and demonstrated self-kindness, mindfulness, and connectedness. Significant gains occurred across all levels of the intervention indicating that benefits initially began within the first 2weeks of treatment followed by continued progression throughout the intervention. When comparing pre-treatment, mid-treatment, and post-treatment means, large effect sizes were observed for each transdiagnostic variable with values-based action exhibiting the largest effect size. Established SUD interventions traditionally display small effect sizes which are short in duration (Lee et al., 2015). Present outcomes support findings indicating superior effect sizes for ACT models compared to established protocols and suggests that CHOPS may also be an effective alternative for SUD. It should be noted that CHOPS assessed transdiagnostic processes as opposed to abstinence rates make comparisons between modalities difficult (Lee et al., 2015).

Previous studies additionally indicate that ACT interventions are prone to *incubation effects* where therapeutic benefits are maintained at follow-up (González-Menéndez et al., 2014). The present study builds upon these findings and suggests that CHOPS demonstrated similar therapeutic gains which were maintained at follow-up compared to both baseline and post-treatment. Effect sizes were largest when comparing baseline and follow-up means. Developing interventions capable of maintaining treatment gains is particularly important in a population where relapse is common.

Recent ACT literature has developed an interest in determining whether ACT interventions also create a longer-term sleeper effect, where therapeutic gains are not only maintained at follow-up but improved upon after therapy completion (Lee et al., 2015). Consistent with Lee et al. (2015), current findings indicated a sleeper effect for psychological inflexibility, suggesting continued benefits at follow-up beyond post-treatment gains. An additional sleeper effect was also observed for mindfulness, a component of self-compassion, indicating that mindful awareness continued increasing after treatment conclusion. Sleeper effects were not observed for values-based action or total self-compassion as were originally expected. Together, these findings indicate that Choice Point ACT may result in more robust outcomes than established protocols.

Because SUD relapse is commonplace, an investigation into long-term benefits of targeting transdiagnostic processes is warranted. Some meta-analyses have found that ACT better maintained abstinence at follow-up when compared to established SUD protocols (Lee et al., 2015). The present study adds to these findings by assessing the relationship between transdiagnostic processes and warning signs of relapse at follow-up. Findings indicate that self-compassion and psychological inflexibility both demonstrated a strong relationship with warning signs of relapse. Participants who reported greater self-compassion indicated fewer relapse signs, while those reporting increased psychological inflexibility indicated greater relapse signs at follow-up. Values-based action and warning signs of relapse were also strongly related at follow-up suggesting that those taking actionable steps toward values additionally exhibited fewer relapse signs. These findings add to a growing body of ACT literature and suggest that Choice Point ACT may also result in better long-term abstinence rates than established protocols for SUD. Further, increasing psychological flexibility, values-based action, and self-compassion have the potential to reduce relapse rates in the long term (Lanza et al., 2014).

Attendance frequency was examined as a measure of treatment adherence. Those with SUD or chronic mental health disorders are 50% less adherent, contributing to relapses and re-hospitalizations (Herbeck et al., 2005; Gaudiano et al., 2012; Moitra and Gaudiano, 2016). Those with co-occurring presentations are at even greater risk (Herbeck et al., 2005). Present findings indicate that 85.1% of those who completed the study missed a total of 0–1 sessions, suggesting strong treatment adherence among those participants. This includes periods of detox, which are notoriously challenging times for therapy engagement.

The relationship between transdiagnostic processes and treatment adherence was also investigated to determine if transdiagnostic approaches, such as CHOPS, are viable methods for targeting adherence. Psychological inflexibility demonstrated a moderate inverse relationship with treatment adherence indicating that participants exhibiting greater psychological inflexibility were also less treatment compliant. This suggests that increasing psychological flexibility could positively impact adherence in SUD, chronic mental health, and co-occurring populations. Values-based action and self-compassion were not significantly related to treatment adherence suggesting a lack of meaningful relationship between these constructs.

This study is subject to several limitations for consideration. First, given the lack of control group and randomization, we cannot rule out the possibility of confounding variables influencing the results of the study. Participants were also exposed to a multitude of therapies outside of the study including Dialectical Behavior Therapy (DBT), CBT, alternative therapies, and medication therapy. It is also possible that admission into a residential program was itself behaviorally activating and provided motivation for treatment adherence. Future studies should include a comparison group in order to control for extraneous variables and assess for motivational levels or stages of change. It also may be difficult to determine if CHOPS specific activities particularly influenced outcomes as traditional ACT metaphors, common Choice Point activities, and novel CHOPS interventions were utilized.

Second, because the population sample consisted of residents in an inpatient facility, threats to external validity exist. It is unclear the extent to which findings can be generalized to outpatient settings, nonclinical settings, or other inpatient settings. Additionally, the population sample was 87% Caucasian making it more difficult to generalize findings across race and ethnicity. Future studies should aim to be more inclusive and generalizable.

Third, while 3-month follow-up data suggest that therapeutic gains were maintained and improved upon in some cases, 12-month follow-up is warranted. Studies supporting long-term benefits of ACT do exist; however, further analysis is needed (González-Menéndez et al., 2014; Lanza et al., 2014). Follow-up data collection was challenging due to changing contact information and places of residence. Correspondence conducted through phone, email, Internet, and in-person conference was minimally successful, resulting in small sample sizes and underpowered *t*-tests. It is possible that lack of power resulted in type-II error, negatively impacting the ability to find true sleeper effects for values-based action and self-compassion. Underpowered correlational analyses were also a consequence of small sample size which may have erroneously contributed to nonsignificant findings when examining relationships with treatment adherence. Additionally, abstinence rates were not assessed at follow-up. Future studies should assess abstinence especially with regards to how it relates to transdiagnostic processes.

Fourth, using a repeated measures design, such as an ANOVA or a paired sample *t*-test, also has drawbacks. While a repeated measures design allows for greater power and smaller sample sizes, participants are also more susceptible to carryover effects (Cleophas, 1999). This study additionally relied exclusively on self-report measures. While self-report measures provide an accessible manner of data collection, they are vulnerable to response bias when respondents present themselves in socially desirable ways (McDonald, 2008; Crutzen and Göritz, 2010). It is important to mention that the AWARE Questionnaire was modified for the purpose of assessing warning signs of substance use rather than warning signs specific to alcohol use. Phrasing was minimally amended; however, it is possible that validity was compromised as a result of the revision.

## Conclusion

CHOPS demonstrated preliminary feasibility and acceptability in the treatment of SUD. To our knowledge, this is also the first application of the Choice Point model in an inpatient facility. As expected, main effects were observed in psychological inflexibility, values-based action, and self-compassion, with significant gains occurring across all levels of the intervention. Therapeutic benefits were maintained at follow-up for all three transdiagnostic processes and improved upon in the areas of psychological inflexibility and mindfulness. Transdiagnostic variables were correlated with warning signs of relapse, while treatment adherence was associated with psychological inflexibility at follow-up.

These findings have important implications for the treatment of SUD and co-occurring disorders. Due to increasingly high attrition rates and limitations of managed care, interventions capable of providing early therapeutic gains is optimal. Because treatment adherence influences outcomes, targeting psychological inflexibility may provide a novel means of improving compliance. Additionally, CHOPS may be an effective alternative model for achieving longer-term abstinence as evidenced by sustained treatment gains and sleeper effect findings. Interventions proficient at maintaining therapeutic benefits while building upon those gains are desirable in a population where relapse is likely to occur.

## Data Availability Statement

The original contributions presented in the study are included in the article/supplementary material, and further inquiries can be directed to the corresponding author.

## Ethics Statement

The studies involving human participants were reviewed and approved by the Lancaster General Health Institutional Review Board. The patients/participants provided their written informed consent to participate in this study.

## Author Contributions

BB and KK collaborated on study design and data collection. BB performed statistical analyses and data interpretation. KK created manuscript tables. All authors were involved in drafting, revising, and approving submission of the manuscript.

## Conflict of Interest

The authors declare that the research was conducted in the absence of any commercial or financial relationships that could be construed as a potential conflict of interest.

## Publisher’s Note

All claims expressed in this article are solely those of the authors and do not necessarily represent those of their affiliated organizations, or those of the publisher, the editors and the reviewers. Any product that may be evaluated in this article, or claim that may be made by its manufacturer, is not guaranteed or endorsed by the publisher.
